# D-Dimer: Not Just an Indicator of Venous Thrombosis but a Predictor of Asymptomatic Hematogenous Metastasis in Gastric Cancer Patients

**DOI:** 10.1371/journal.pone.0101125

**Published:** 2014-07-01

**Authors:** Dongmei Diao, Zhe Wang, Yao Cheng, Hao Zhang, Qi Guo, Yongchun Song, Kun Zhu, Kang Li, Di Liu, Chengxue Dang

**Affiliations:** 1 Department of Oncology Surgery, First Affiliated Hospital of Xi'an Jiaotong University, Xi'an, China; 2 Department of Thoracic Surgery, First Affiliated Hospital of Xi'an Jiaotong University, Xi'an, China; 3 Department of Oncology Surgery, Second Affiliated Hospital of Xi'an Jiaotong University, Xi'an, China; Deutsches Krebsforschungszentrum, Germany

## Abstract

**Background:**

Plasma D-dimer levels have been shown to be high in advanced tumor stage patients and can be used to predict clinical outcome in cancer patients. As most advanced tumor stage patients exhibit asymptomatic metastasis, which contributes to early tumor recurrence after surgery, we hypothesized that plasma D-dimer levels can be used to predict patients with potential metastasis.

**Methods:**

We enrolled 1042 primary gastric cancer patients in three multiple cancer centers in Northwest China and examined plasma D-dimer levels using the latex-enhanced immunoturbidimetric assay (LEIA) method. Plasma D-dimer levels were compared with the clinicopathological characteristics in this large-scale case-control study with follow up. We also performed regular follow-up studies for 395 patients to analyze the 2-year survival rate and early tumor recurrence.

**Results:**

In this large-scale clinical study, we found that plasma D-dimer levels were increased in patients with distant metastasis and especially hematogenous metastasis patients. The cut-off value of the D-dimer levels was determined to be 1.5 mg/ml based on the ROC curve, and the sensitivity and specificity for metastasis prediction were 61.9% and 86.6%, respectively. Additionally, patients with high D-dimer levels displayed early tumor recurrence and poor outcome during the follow-up study.

**Conclusion:**

Plasma D-dimer may represent an easy to measure and lower cost marker for the testing of gastric cancer patients to predict asymptomatic hematogenous metastasis.

## Introduction

Hematogenous metastasis means blood borne metastasis is the most critical complication of malignancy; however, our understanding of this phenomenon remains incomplete [1]. Once malignant cells enter into the circulation, they must be able to survive several stresses, including physical damage from hemodynamic shear forces and immune-mediated cell killing [2], [3]. It has been suggested that the formation of platelet-fibrin-tumor cell aggregates may play a causal role in endothelial adhesion and metastatic potential [4]. Deposition of fibrin within adherent tumor cell-platelet aggregates can be detected as early as 5 minutes after tumor cell inoculation and persists for more than 9 hours [5]. Fibrinogen is a crucial source of bio-available fibrin to tumor cells in the vasculature, which is necessary for tumor cells in the vasculature, tumor cell extravasation and metastasis formation [2], [3]. Thus, plasma D-dimer may be involved in the promotion of a metastatic phenotype in the bloodstream.

D-dimer, which is a stable end product of the degradation of cross-linked Fibrin, results from enhanced Fibrin formation and fibrinolysis. D-dimer levels are widely used to detect patients with suspected disseminated intravascular coagulation (DIC), thromboembolic events, and myocardial infarction [6]–[8]. Recently, it has been reported that increased D-dimer levels correlate with malignancy [9], [10]. Additionally, a number of studies have reported that D-dimer levels are associated with tumor stage, tumor prognosis, lymph node involvement, and overall survival in patients with solid tumors, such as lung cancer [11], [12], breast cancer [13], esophageal cancer [10], gastric cancer [14], colon cancer [15], and gynecological malignancies [16]. Plasma D-dimer levels were shown to be high in advanced tumor stage patients and can be used to predict clinical outcome in cancer patients [17], [18], [19], [20], [21]. Moreover, most of the advanced tumor stage patients harbored asymptomatic hematogenous metastases. We hypothesized that plasma D-dimer levels might be a useful clinical marker for hematogenous metastasis in malignancy.

Based on previous studies, we designed this study to examine the effectiveness of managing suspected metastasis using plasma D-dimer tests in clinical cancer patients. In this study, we established a prospective clinical trial in three multiple clinical cancer centers in Northwest China. Gastric cancer (GC) is one of the most common malignant tumors reported in Asia; however, the prognosis of patients with advanced disease remains very poor [22], [23]. A variety of clinical and biological variables have been proposed as prognostic factors for GC, but the precise molecular mechanism underlying and predicting the development and progression of GC in clinical use remains unclear [24]. Therefore, we examined the levels of plasma D-dimer, fibrinogen (FIB), fibrin degradation products (FDP), and the tumor marker carcinoembryonic antigen (CEA), which is also commonly used in GC clinics in primary GC patients. We evaluated the association of these findings with clinical and pathological factors as well as disease-free survival (DFS) and overall survival (OS) outcome. Furthermore, we examined plasma D-dimer levels in other malignancies that easily metastasize, including melanoma and pancreatic cancer, to estimate the clinical use D-dimer tests.

## Materials and Methods

### 1. Study design

This study was based on the hypothesis that D-dimer levels can be used to detect metastasis in cancer patients. We first verified our hypothesis in a large-scale clinical study. Plasma D-dimer levels were assessed in 1042 primary GC patients and compared with clinical and pathological factors. The effectiveness of managing suspected metastasis based on plasma D-dimer levels in GC patients was evaluated via Receiver operator characteristic (ROC) curve. Finally, we performed a follow-up study with a subset of the GC patients to estimate overall survival (OS) and early tumor recurrence (ETR, recurrence within 2 years) based on high and low D-dimer levels based on the ROC curve. We also verified our conclusion by assessing other solid tumors (melanoma and pancreatic cancer) in our clinical investigation.

### 2. Patients

Primary cancer patients included in the study were diagnosed via pathological examination via endoscopic biopsy and hospitalized in one of three cancer centers (the First Affiliated Hospital of Xi'an Jiaotong University, the Second Affiliated Hospital of Xi'an Jiaotong University and Xijing Hospital of the Fourth Military Medical University) between January 1^st^ 2009 and January 1^st^ 2012. Patients with a history of venous thrombosis or anticoagulation therapy, cardiovascular and cerebrovascular disease, acute or chronic inflammatory disease, previous malignancy, or previous anticancer treatment were excluded. After excluding certain patients (a total of 367 patients excluded, for the following reasons: 135 with infection; 94 with previous anticancer treatment; 79 with cardiac-cerebral vascular disease, and 59 with other excluded reasons, and the characters of those excluded were similar to cases included after statistical analyses.), 1123 patients (i.e., 1042 GC, 44 pancreatic cancer, 37 melanoma patients) were enrolled in our study. Additionally, 50 health volunteers, 31 patients with gastric polyps (GP) and 35 gastric stromal tumor (GST) patients were enrolled in this study at the same time. Based on the results of the clinical evaluation, 1042 GC patients were included, 787 patients underwent complete resection with negative magins (R0 resection), 19 patients underwent gastric resection and regional lymph node resection but with microscopic residual disease(R1 resection); 172 underwent palliative operation and 64 patients only received exploratory laparotomy. A regular follow-up was performed for 395 out of 420 GC patients who were hospitalized at the First Affiliated Hospital of Xi'an Jiaotong University, and the follow-up for all cases was terminated in Dec. 2012. The first end-point refers to patients who were diagnosed with tumor recurrence, and the second point was followed for >2 years or until death. Tumor stages were recorded using the classification guidelines of the American Joint Committee on Cancer (AJCC). Metastasis was usually detected by imaging detection (Computed Tomography(CT) scan, an ultrasonic B scan, or Positron emission tomography–computed tomography (PET-CT), bone marrow scan) and pathology detection. Lymphovascular invasion was assessed in resection specimen, and discriminate between lymphatic or blood vessel infiltration was by immunohistochemistry (IHC), blood vessel was stained by CD34 in our study. For GC patients, follow up included a complete history and physical examination every 3–6 months for 1 to 2 years, CT scan of the chest and abdomen, an ultrasonic B scan of the abdomen (live and adrenal glands), bone marrow scan and endoscopy biopsy to exclude recurrence and metastasis. GC patients with advanced tumour stage were treated with the same adjunctive treatment (5-fluorouracil, oxaliplatin and folinic acid; 4 times; once a month) after surgery in this hospital while a large number of GC patients with advanced tumour stages did not proceed adjunctive treatment as can't bear the high cost of the treatment. Our study was approved by the Conduct of Human Ethics Committee of Xi'an Jiaotong University, and written informed consent was provided by all patients.

### 3. D-dimer assays

Venous blood samples were collected in tubes with sodium citrate for the measurement of D-dimer levels. Plasma CEA levels in GC patients were assayed using the ELISA method. In the clinical study, the D-dimer, FDP, and FIB levels were assayed via latex-enhanced immunoturbidimetric assay as described in our previous study [25].

### 4. Statistical analysis

Data analysis was performed using SPSS 13.0 Software (SPSS, Chicago, IL, USA). As the D-dimer levels were not normally distributed, the results of the D-dimer level tests are reported as median (M) and quartile range (Q). Statistical tests were performed using the Mann-Whitney U test and the Kruskal-Wallis H test. For univariate analysis, Spearman Correlation analysis was employed. Multiple linear regression models were used to identify independent variables that correlated with plasma D-dimer levels. The ROC curve was used to estimate the effectiveness of D-dimer levels in predicting metastasis. The Kaplan-Meier method was used to estimate the distribution of survival curves, and log-rank tests were used to compare the distributions between groups. Cox proportional hazards regression analysis was performed to identify independent variables that correlated with patient survival and DFS. P<0.05 was considered statistically significant.

## Results

### 1. Patient data and D-dimer levels for different groups

**Figure 1:**
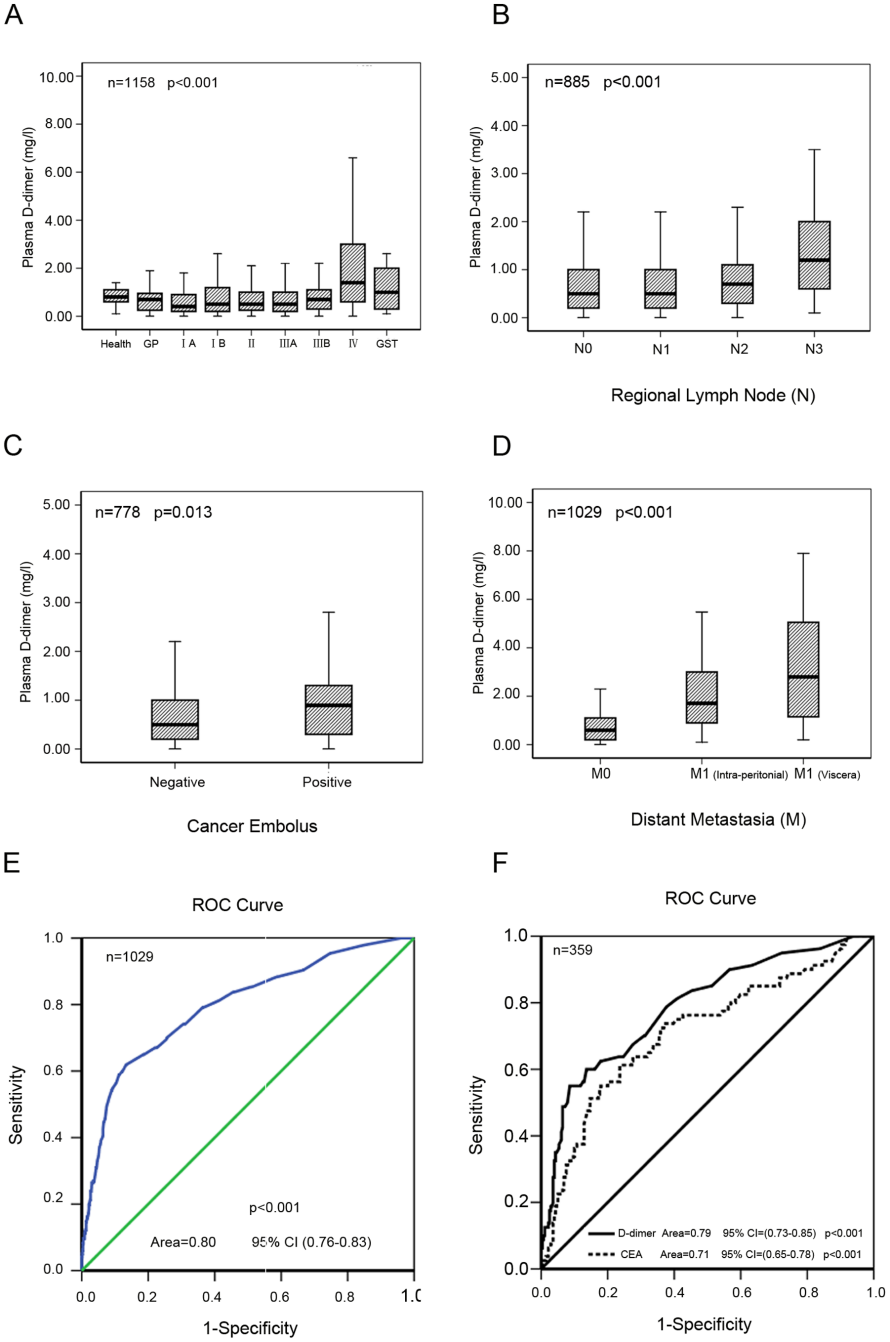
Plasma D-dimer levels in GC patients. A, Plasma D-dimer levels were significantly increased in stage IV GC patients (p<0.001) but did not show a significant difference between other groups. B, C, D, Plasma D-dimer levels were significantly higher in patients with N3 lymph node invasion (P<0.001), positive vascular cancer embolus in resected GC tissue samples (P = 0.013) and distant metastasis (P<0.01). E, F, ROC curve for metastasis detection via the plasma D-dimer test (P<0.001, area  = 0.80, 95%CI (0.76–0.83)) and CEA levels (P<0.001, area  = 0.71, 95%CI (0.65–0.78)).

Basic patient data (age and gender) and plasma D-dimer levels (median and 25th–75th percentile) are listed in Table S1. In total, 1042 GC patients (837 men and 205 women, aged 22–88 years) were included in the study; other GC patients were excluded for certain reasons, as shown in Table S1. The control groups included 50 health volunteers (25 men and 25 women, aged 36–84 years, median D-dimer levels is 0.80 mg/ml), 31 patients with GP (10 men and 21 women, aged 36–75 years, median D-dimer levels is 0.70 mg/ml) and 35 GST patients (23 men and 12 women, aged 36–75 years, median D-dimer levels is 1.0 mg/ml). Plasma D-dimer levels were tested among the various groups, and the results are shown in Table S1. The results display a significant increase D-dimer levels among the stage IV GC group (median 1.4 mg/l) compared with the other groups (p<0.001), while plasma D-dimer levels did not display a significant difference in other groups (Figure 1A).

### 2. Correlation between plasma D-dimer levels and associated factors

The correlation between plasma D-dimer levels and associated factors is shown in Table 1. The results of the correlation analysis via multiple linear regression model show that plasma D-dimer levels only correlated with lymph node invasion (r = 0.167; p = 0.01), the presence of vascular cancer emboli in tissue specimens (r = 0.367; p = 0.043) and distant metastasis (r = 0.466; p = 0.007); the results are shown in Table 1 and Figures 1B–1D.

**Table 1:** Association between D-dimer levels (mg/l) and Clinicopathological Features in Patients with Gastric Cancer (n = 1042).

Variables	No. of cases	Median	25^th^–75^th^ percentile	*R/P (Uni)*	*B/P (Multi)*
Patients	1042	0.70	1.085		
Gender				0.098/0.002**	0.274/0.053
Male	837	0.7	0.30–1.30		
Female	205	0.9	0.40–1.65		
Age				0.157/<0.001**	0.007/0.171
≤60	518	0.60	0.20–1.10		
>60	524	0.89	0.40–1.30		
Histological grade				0.108/<0.001**	−0.027/0.743
G1	55	0.50	0.20–1.27		
G2	423	0.60	0.20–1.20		
G3	508	0.90	0.40–1.50		
G4	28	0.40	0.20–0.80		
GX	28	0.90	0.30–2.07		
Tumor invasion				0.276/<0.001**	0.049/0.586
T1	84	0.50	0.20–0.90		
T2	99	0.50	0.20–1.19		
T3	644	0.60	0.30–1.10		
T4	192	1.30	0.60–2.77		
Lymph node invasion				0.220/<0.001**	0.167/0.01*
N0	279	0.50	0.20–1.00		
N1	311	0.50	0.20–1.00		
N2	180	0.70	0.30–1.10		
N3	115	1.20	0.20–2.00		
Distant metastasis	1028			0.409/<0.001**	0.466/0.007**
M0	832	0.60	0.20–1.10		
M1	109	1.70	0.85–3.05		
M2	88	2.80	1.12–5.07		
Cancer embolus				0.089/0.013*	0.367/0.043*
Negative	696	0.50	0.20–1.00		
Positive	82	0.89	0.30–1.30		

Footnote: R indicates the correlation coefficient. P (Uni) indicate that the p-value was analyzed via Spearman Correlation analysis was employed; B means partial regression confidence. P (Multi) indicate that the p-value was analyzed via multivariate linear regression models, and all covariates are inclouded.

*indicates p-value <0.05 (2-tailed). R indicates the correlation coefficient.

**indicates p-value <0.01 (2-tailed). Histological grade(G): G1 well differentiated; G2 Moderately differentiated; G3 Poorly differentiated; G4 Undifferentiated; G5 should be Gx means grade cannot be assessed. M1 means intra-peritoneal metastasis; M2 means visceral metastasis.

Plasma FDP levels displayed a linear association with D-dimer (R^2^ = 0.853, p<0.001) (Figure S1A). However, no linear association was found between FIB levels and D-dimer levels (R^2^ = 0, p = 0.61) (Figure S1B). These results were consistent with the in vivo results. Additionally, CEA levels showed a weak linear association with D-dimer levels in GC patients (R^2^ = 0.056, p<0.001) (Figure S1C).

### 3. Effectiveness of managing suspected metastasis based on plasma D-dimer data

Plasma D-dimer levels were markedly increased in distance metastasis patients compared with control patients, especially in patients with hematogenous visceral metastasis, as shown in Figure 1D. Plasma D-dimer levels were elevated in TNM stage IV patients and N3 lymph node invasion patients; most of these patients had distant metastases or potential metastasis. Therefore, we hypothesized that plasma D-dimer levels can be used to detect metastasis. Receiver operator characteristic (ROC) curves for the D-dimer assay in the diagnosis of metastasis are shown in Figure 1E. The cut-off values for the D-dimer levels was determined to be 1.5 mg/ml based on the ROC curve, and the sensitivity and specificity for metastasis prediction were 61.9% and 86.6%, respectively. ROC curve for metastasis detection via the plasma D-dimer test (P<0.001, area  = 0.80, 95%CI (0.76–0.83)) and CEA levels in GC patients in the present study, as shown in Figure 1F.

### 4. Plasma D-dimer levels, OS and DFS

**Figure 2:**
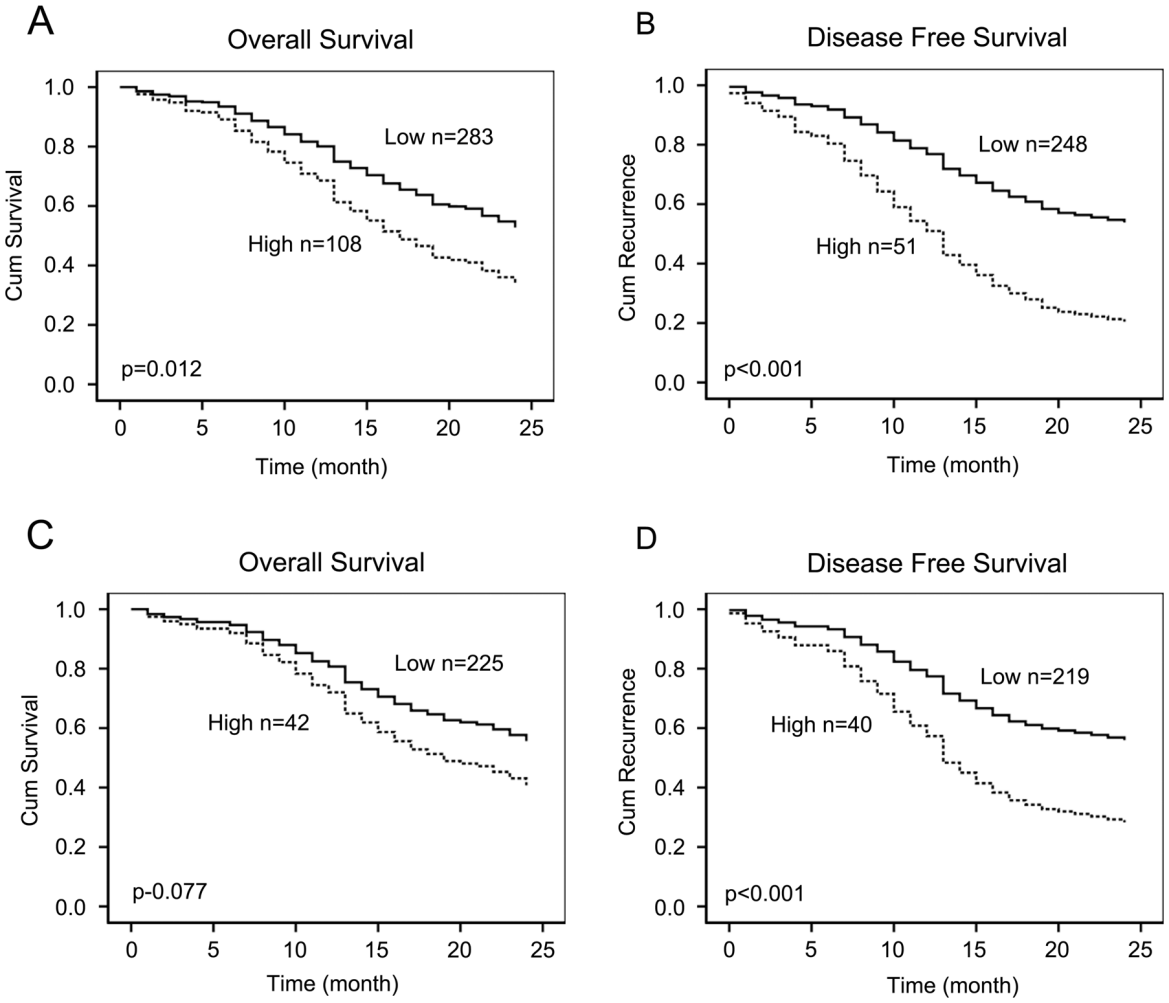
Cox proportional hazards regression model analysis of the two-year survival and disease-free survival in 395 GC patients. A, B, After defining the high and low levels of plasma D-dimer by 1.5 mg/l based on the ROC results. Overall survival and DFS were reduced in all GC patients with high D-dimer levels (P<0.001, P<0.001, respectively). C, D, In stageI–III GC patients, survival and DFS were reduced in patients with high D-dimer levels (P = 0.042, P<0.001, respectively).

In total, 395 patients were successfully examined during follow-up, 292 patients underwent R0 resection, 4 patients underwent R1 resection, 65 underwent palliative operation and 34 patients only received exploratory laparotomy, 93 GC patients with advanced tumour stage were treated with the same adjunctive treatment. We defined the high and low levels according to the plasma D-dimer levels at 1.5 mg/ml based on the ROC curve. During the follow-up, 177 of the 391 patients died; the 2 year survival was 54.7% overall. For GC patients with high D-dimer levels, the survival time was short based on the log-rank survival analysis. Additionally, survival time correlated with tumor invasion (p = 0.002), lymph node invasion (p<0.001), surgery method (p = 0.025), vascular cancer emboli (p = 0.026) and additional treatment (p = 0.026), based on the log-rank survival test. However, survival time was not influenced by patient gender (p = 0.778), age (p = 0.336) or histological grade (p = 0.39). Based on the Cox proportional hazards regression analysis, survival was influenced by D-dimer levels (p = 0.012, Risk ratio = 1.7) (Figure 2A), lymph node invasion (p = 0.011, Risk ratio = 1.33) and additional treatment (p = 0.045, Risk ratio = 0.61). The results are shown in Table 2.

**Table 2:** Prognostic variables for overall survival and disease free survival in GC patients.

Prognostic	Overall Survival			Disease Free Survival		
Variable	P(uni)	P(multi)	RR (95% CI)	P(uni)	P(multi)	RR (95% CI)
D-dimer	<0.001**	0.012*	1.7(1.16, 2.56)	<0.001**	<0.001**	2.44 (1.63, 3.65)
Age	0.366			0.418		
Gender	0.778			0.967		
Grade	0.39			0.799		
T	0.002**	0.464	1.13(0.82, 1.57)	0.007**	0.38	1.15(0.84, 1.58)
N	<0.001**	0.011*	1.33(1.07, 1.65)	<0.001**	0.033*	1.26 (1.02, 1.57)
M	0.081					
Surgery	0.025*	0.605	1.27(0.51, 3.19)	0.153		
Cancer Embolus	0.026*	0.293	1.37(0.76, 2.47)	0.046*	0.197	1.48(0.81, 2.69)
Additional Treatment	0.026*	0.045*	0.61(0.37, 0.99)	0.403		

Footnote: P (Uni) indicate that the p-value was analyzed via Kaplan-Meier method; P (Multi) indicate that the p-value was analyzed via Cox proportional hazards regression models and all significant covariates from the univariate model are inclouded.

*indicates p-value <0.05 (2-tailed).

**indicates p-value <0.01 (2-tailed). RR, Risk Ratio; CI, confidence interval.

Disease-Free Survival (DFS) is the most crucial clinical event associated with poor prognosis of surgical GC before death and might be regarded as more important than mortality. In this study, 203 of the 382 patients found tumor recurrence within 2 years; the recurrence rate was 53.1% overall. In order to better interview the significance of plasma d-dimer, we exclude 83 diagnosed distance metastasis GC patients at the beginning, 140 of 299 GC patients found tumor recurrence whin 2 years with the recurrence rate is 46.8%. A significant difference in DFS was observed between the GC patients with high D-dimer levels and those with low levels. Univariate analysis showed that elevated plasma D-dimer levels, tumor invasion, lymph node invasion, and cancer emboli were significant predictive factors for DFS; however, multivariate analysis showed that only elevated plasma D-dimer levels (p<0.001, risk ratio = 2.44) (Figure 2B) and lymph node invasion (p = 0.033, risk ratio = 1.26) were significant independent risk factors in the Cox's proportional hazards model for time to DFS as listed in Table 2.

Of the 269 patients with TNM stage I, II, or III, 105 of 267paitents died within 2 years with the survival rate is 60.7%, 114 of 259 patients found tumor recurrence within 2 years with the recurrence rate is 44.0%. The survival time was short for patients with high D-dimer levels, but the difference was not statistically significant based on Cox proportional hazards regression model (Figure 2C), Interestingly, DFS was short in patients with high D-dimer levels (p<0.001, Risk ratio = 2.7) (Figure 2D) according to both log-rank and Cox regression analyses as shown in Table 3.

**Table 3:** Prognostic variables for overall survival and disease free survival in Stage I–III GC patients.

Prognostic	Overall Survival			Disease Free Survival		
Variable	P(uni)	P(multi)	RR (95% CI)	P(uni)	P(multi)	RR (95% CI)
D-dimer	0.042*	0.077	1.53(0.95, 2.45)	<0.001**	<0.001**	2.17(1.4, 3.38)
Age	0.735			0.733		
Gender	0.844			0.432		
Grade	0.567			0.604		
T	0.051			0.101		
N	0.011*	0.008**	1.37(1.09, 1.74)	0.004**	0.003**	1.41(1.12, 1.77)
Surgery	0.753			0.897		
Cancer Embolus	0.299			0.297		
Additional Treatment	0.041*	0.0587	0.61(0.37, 1.02)	0.319		

Footnote: P (Uni) indicate that the p-value was analyzed via Kaplan-Meier method; P (Multi) indicate that the p-value was analyzed via Cox proportional hazards regression models and all significant covariates from the univariate model are inclouded.

*indicates p-value <0.05 (2-tailed).

**indicates p-value <0.01 (2-tailed). RR, Risk Ratio; CI, confidence interval.

### 5. Plasma D-dimer levels in other solid tumors

**Figure 3:**
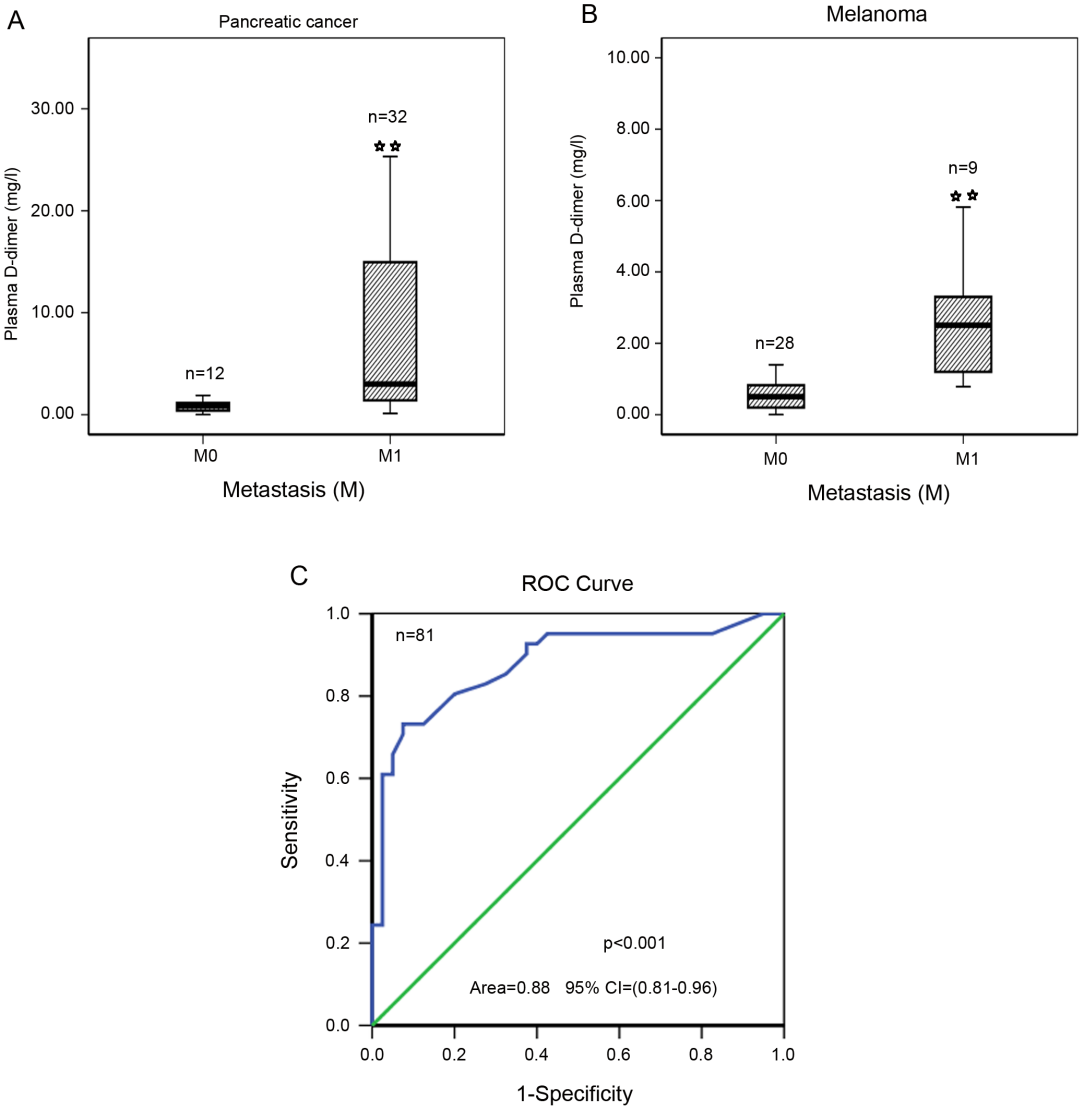
Plasma D-dimer levels in other solid tumors. A, Plasma D-dimer levels were increased in metastasis pancreatic cancer patients (p<0.001). B, Plasma D-dimer levels were increased in metastasis melanoma patients (p<0.001). C, ROC curve for metastasis detection based on the plasma D-dimer levels (P<0.001, Area = 0.88, 95%CI (0.81–0.96)). * indicates p-value <0.05 (2-tailed). ** indicates p-value <0.01 (2-tailed).

In our previous study, we found that D-dimer levels were significantly higher in TNM stage esophageal cancer patients [25]. We also examined plasma D-dimer levels in 44 pancreatic cancer patients (31 metastasis) and 37 melanoma patients (9 metastasis) and found that plasma D-dimer levels were notably elevated in pancreatic cancer and melanoma patients with metastasis (Figures 3A, 3B). The cut-off value for D-dimer levels was determined to be 1.5 mg/ml based on the ROC curve (Figure 3C), and the sensitivity and specificity for metastasis prediction were 73.2% and 92.5%, respectively. If the cut-off value for D-dimer levels is determined to be 1.5 mg/ml, the positive predictive value is 90.9% and the negative predictive value is 77.1%.

## Discussion

Metastasis remains the major cause of cancer treatment failure. Coagulation has long been known to facilitate metastasis [5], [26], [27], [28], [29]. In clinical studies, we found that both plasma D-dimer levels were significantly elevated in metastasis GC patients, especially in patients with hematogenous visceral metastasis, and D-dimer levels correlated with vascular cancer emboli in resected tissue samples. GC patients with elevated D-dimer levels displayed decreased survival. Importantly, DFS was significantly decreased in GC patients with high D-dimer levels. Interestingly, patients with TNM I, II, and III stages with elevated plasma D-dimer levels showed a higher rate of tumor recurrence compared with control patients, although the Cox regression hazard model showed that OS did not differ based on plasma D-dimer levels. These findings led us to believe that plasma D-dimer is a predictive marker of asymptomatic metastasis, which is associated with poor outcomes in GC patients.

Analyzing fibrinogen-deficient mice, Palumbo et al. have shown that fibrinogen plays a role in adhesion and survival of tumor cells rather than growth of tumor cells [30], [31]. At the beginning of the present study, we hypothesized that coagulation factor can be used to detect metastasis in the clinical setting; therefore, we tested fibrinogen in association with factor D-dimer levels and FDP. We found that plasma fibrinogen did not markedly vary in GC patients. Although it is an important factor in Circulating Tumor Cells (CTC) survival and metastasis, it is not available in clinical metastasis tests. However, plasma D-dimer levels showed an advantage in this assessment. FDP involves D-dimer and degradation of fibrinogen, it was primarily influenced by D-dimer levels, as indicated by their linear association detected in the present study.

The identification of novel molecular markers for the prediction of tumor metastasis will contribute to the development of better strategies for patient management. Furthermore, in our clinical study, we found that elevated plasma D-dimer levels were an independent risk factor associated with metastasis, vascular cancer emboli, and disease-free survival (tumor recurrence) and hence contribute to poor survival in patients. We found elevated plasma D-dimer levels not only in metastasis patients but also in some stage IV patients, N3 lymph node invasion patients without obvious metastasis and some stage I, I I, and I I I patients. After a short follow-up study, we found that most of these patients showed tumor recurrence with hematogenous metastasis. The elevation of plasma D-dimer levels is an interesting phenomenon associated with the underlying micro-tumor metastasis of most of these patients, but it cannot be detected via current detection methods, in spite of the fact that elevated plasma D-dimer levels were first detected in these patients. Plasma D-dimer levels represent a better predictor of metastasis in GC patients. Although tumor markers such as CEA are widely used for the follow-up of patients with gastrointestinal cancers, their lack of sensitivity remains an issue. We also found that circulating D-dimer levels are better predictors of metastasis, OS and DFS than CEA levels in GC patients.

Due to the inaccuracy of CT and other modalities for the detection of asymptomatic hematogenous metastasis, it is often difficult to determine whether patients are eligible for potentially curative resection via current generation CT scanning. Current imaging modalities and tumor markers are sometimes useless for monitoring therapy. Measurement of D-dimer levels may be a useful diagnostic tool to predict asymptomatic hematogenous metastasis.

In some high malignancy diseases, such as pancreatic cancer and melanoma, we found that D-dimer levels were remarkably high in metastasis patients in this study. In our previous study, we found that this factor was elevated in metastasis patients with lung cancer and esophageal cancer [25]. Therefore, we hypothesize that this factor can be used in clinical tests for a variety of tumor types.

During the follow-up study, there are some insufficient exists in our present study. The first one is how many of the patients developed thrombotic complications is not cleared. Patients were followed systematically (refer to NCCN guidelines for GC). For asymptomatic patients, follow up included a complete history and physical examination, CT scan of the chest and abdomen, an ultrasonic B scan of the abdomen (live and adrenal glands), bone marrow scan and endoscopy biopsy to exclude recurrence and metastasis. But additional tests to evaluate thrombus has not been included unless clinical symptoms detected during the routine follow up. As long as 3 of 395 patients with symptomatic thrombus, while in other patients, as hypercoagulability exist in most of the cancer patients, so asymptomatic thrombus and micro-thrombus can not be excluded. The second one is the elevation of plasma D-dimer in metastasis patients is not so high as it was test in VTE patients. Plasma D-dimer levels in metastasis patients were increased approximately 2-fold and therefore were not as high as observed in VTE patients. The sensitivity was not sufficiently high, considering patient heterogeneity, and it was easily influenced by other factors, such as infection.

In summary, we described the levels of plasma D-dimer, which can reflect enhanced fibrin formation and fibrinolysis hyper-coagulability in a large number of GC patients. Plasma D-dimer levels were high in metastasis GC patients, although the sensitivity and specificity for metastasis prediction were not sufficiently high. However, considering that the test is low cost and easy to perform, it should be considered for the testing of cancer patients, especially in developing countries.

## Supporting Information

Figure S1 **The relationship between plasma D-dimer levels and FDP, FIB and CEA.** A, B, Plasma D-dimer levels showed a linear relation with plasma FDP levels but not with plasma FIB levels. C, Plasma D-dimer levels correlated with plasma CEA levels, although they did not display a linear relation.
(TIF)

Table S1 **Patient characteristics and plasma D-dimer (mg/l) levels in different groups.**
(DOC)

## References

[1] Gupta GP, Massague J (2006) Cancer metastasis: building a framework. Cell 127: 679–695. 17110329 10.1016/j.cell.2006.11.001

[2] Biggerstaff JP, Weidow B, Vidosh J, Dexheimer J, Patel S, et al (2006) Soluble fibrin inhibits monocyte adherence and cytotoxicity against tumor cells: implications for cancer metastasis. Thromb J 4: 12. 16925817 10.1186/1477-9560-4-12 PMC1564130

[3] Palumbo JS, Talmage KE, Massari JV, La Jeunesse CM, Flick MJ, et al (2005) Platelets and fibrin(ogen) increase metastatic potential by impeding natural killer cell-mediated elimination of tumor cells. Blood 105: 178–185. 15367435 10.1182/blood-2004-06-2272

[4] Gay LJ, Felding-Habermann B (2011) Contribution of platelets to tumour metastasis. Nat Rev Cancer 11: 123–134. 21258396 10.1038/nrc3004 PMC6894505

[5] Im JH, Fu W, Wang H, Bhatia SK, Hammer DA, et al (2004) Coagulation facilitates tumor cell spreading in the pulmonary vasculature during early metastatic colony formation. Cancer Res 64: 8613–8619. 15574768 10.1158/0008-5472.CAN-04-2078

[6] Ay C, Vormittag R, Dunkler D, Simanek R, Chiriac AL, et al (2009) D-dimer and prothrombin fragment 1+2 predict venous thromboembolism in patients with cancer: results from the Vienna Cancer and Thrombosis Study. J Clin Oncol 27: 4124–4129. 19636003 10.1200/JCO.2008.21.7752

[7] Dompmartin A, Ballieux F, Thibon P, Lequerrec A, Hermans C, et al (2009) Elevated D-dimer level in the differential diagnosis of venous malformations. Arch Dermatol 145: 1239–1244. 19917952 10.1001/archdermatol.2009.296 PMC5561655

[8] Righini M, Perrier A, De Moerloose P, Bounameaux H (2008) D-Dimer for venous thromboembolism diagnosis: 20 years later. J Thromb Haemost 6: 1059–1071. 18419743 10.1111/j.1538-7836.2008.02981.x

[9] Lippi G, Franchini M, Biasiutti C, Dellagiacoma G, Salvagno GL, et al (2007) Increased D-dimer value and occult cancer in the absence of detectable thrombosis. Haematologica 92: e53–55. 17562593 10.3324/haematol.12237

[10] Tomimaru Y, Yano M, Takachi K, Kishi K, Miyashiro I, et al (2006) Plasma D-dimer levels show correlation with number of lymph node metastases in patients with esophageal cancer. J Am Coll Surg 202: 139–145. 16377507 10.1016/j.jamcollsurg.2005.08.008

[11] Altiay G, Ciftci A, Demir M, Kocak Z, Sut N, et al (2007) High plasma D-dimer level is associated with decreased survival in patients with lung cancer. Clin Oncol (R Coll Radiol) 19: 494–498. 17513096 10.1016/j.clon.2007.04.002

[12] Antoniou D, Pavlakou G, Stathopoulos GP, Karydis I, Chondrou E, et al (2006) Predictive value of D-dimer plasma levels in response and progressive disease in patients with lung cancer. Lung Cancer 53: 205–210. 16769149 10.1016/j.lungcan.2006.03.015

[13] Batschauer AP, Figueiredo CP, Bueno EC, Ribeiro MA, Dusse LM, et al (2010) D-dimer as a possible prognostic marker of operable hormone receptor-negative breast cancer. Ann Oncol 21: 1267–1272. 19880435 10.1093/annonc/mdp474

[14] Kwon HC, Oh SY, Lee S, Kim SH, Han JY, et al (2008) Plasma levels of prothrombin fragment F1+2, D-dimer and prothrombin time correlate with clinical stage and lymph node metastasis in operable gastric cancer patients. Jpn J Clin Oncol 38: 2–7. 18258711 10.1093/jjco/hym157

[15] Kilic M, Yoldas O, Keskek M, Ertan T, Tez M, et al (2008) Prognostic value of plasma D-dimer levels in patients with colorectal cancer. Colorectal Dis 10: 238–241. 17868411 10.1111/j.1463-1318.2007.01374.x

[16] Mirshahi SS, Pujade-Lauraine E, Soria C, Mirshahi M, Fretault J, et al (1992) D-dimer and CA 125 levels in patients with ovarian cancer during antineoplastic therapy. Prognostic significance for the success of anti-cancer treatment. Cancer 69: 2289–2292. 1562974 10.1002/1097-0142(19920501)69:9<2289::aid-cncr2820690914>3.0.co;2-a

[17] Ay C, Dunkler D, Pirker R, Thaler J, Quehenberger P, et al (2012) High D-dimer levels are associated with poor prognosis in cancer patients. Haematologica 97: 1158–1164. 22371182 10.3324/haematol.2011.054718 PMC3409812

[18] Raj SD, Zhou X, Bueso-Ramos CE, Ravi V, Patel S, et al (2012) Prognostic significance of elevated D-dimer for survival in patients with sarcoma. Am J Clin Oncol 35: 462–467. 21654313 10.1097/COC.0b013e31821d4529

[19] Stender MT, Larsen TB, Sorensen HT, Thorlacius-Ussing O (2012) Preoperative plasma D-dimer predicts 1-year survival in colorectal cancer patients with absence of venous thromboembolism (VTE): a prospective clinical cohort study. J Thromb Haemost 10: 2027–2031. 22900573 10.1111/j.1538-7836.2012.04887.x

[20] Tas F, Ciftci R, Kilic L, Bilgin E, Keskin S, et al (2012) Clinical and prognostic significance of coagulation assays in melanoma. Melanoma Res 22: 368–375. 22889867 10.1097/CMR.0b013e328357be7c

[21] Yamamoto M, Yoshinaga K, Matsuyama A, Iwasa T, Osoegawa A, et al (2012) Plasma D-dimer level as a mortality predictor in patients with advanced or recurrent colorectal cancer. Oncology 83: 10–15. 22722426 10.1159/000338329

[22] Jemal A, Siegel R, Ward E, Hao Y, Xu J, et al (2009) Cancer statistics, 2009. CA Cancer J Clin 59: 225–249. 19474385 10.3322/caac.20006

[23] Kamangar F, Dores GM, Anderson WF (2006) Patterns of cancer incidence, mortality, and prevalence across five continents: defining priorities to reduce cancer disparities in different geographic regions of the world. J Clin Oncol 24: 2137–2150. 16682732 10.1200/JCO.2005.05.2308

[24] Ahn HS, Shin YS, Park PJ, Kang KN, Kim Y, et al (2012) Serum biomarker panels for the diagnosis of gastric adenocarcinoma. Br J Cancer 106: 733–739. 22240791 10.1038/bjc.2011.592 PMC3322950

[25] Diao D, Zhu K, Wang Z, Cheng Y, Li K, et al (2013) Prognostic value of the D-dimer test in oesophageal cancer during the perioperative period. J Surg Oncol 108: 34–41. 23677634 10.1002/jso.23339

[26] Horowitz NA, Blevins EA, Miller WM, Perry AR, Talmage KE, et al (2011) Thrombomodulin is a determinant of metastasis through a mechanism linked to the thrombin binding domain but not the lectin-like domain. Blood 118: 2889–2895. 21788337 10.1182/blood-2011-03-341222 PMC3172805

[27] Labelle M, Begum S, Hynes RO (2011) Direct signaling between platelets and cancer cells induces an epithelial-mesenchymal-like transition and promotes metastasis. Cancer Cell 20: 576–590. 22094253 10.1016/j.ccr.2011.09.009 PMC3487108

[28] van den Berg YW, Osanto S, Reitsma PH, Versteeg HH (2012) The relationship between tissue factor and cancer progression: insights from bench and bedside. Blood 119: 924–932. 22065595 10.1182/blood-2011-06-317685

[29] Zhang W, Dang S, Hong T, Tang J, Fan J, et al (2012) A humanized single-chain antibody against beta 3 integrin inhibits pulmonary metastasis by preferentially fragmenting activated platelets in the tumor microenvironment. Blood 120: 2889–2898. 22879538 10.1182/blood-2012-04-425207 PMC3466970

[30] Palumbo JS, Kombrinck KW, Drew AF, Grimes TS, Kiser JH, et al (2000) Fibrinogen is an important determinant of the metastatic potential of circulating tumor cells. Blood 96: 3302–3309. 11071621

[31] Palumbo JS, Potter JM, Kaplan LS, Talmage K, Jackson DG, et al (2002) Spontaneous hematogenous and lymphatic metastasis, but not primary tumor growth or angiogenesis, is diminished in fibrinogen-deficient mice. Cancer Res 62: 6966–6972. 12460914

