# Supplementary figures and images for: D-Dimer: Not Just an Indicator of Venous Thrombosis but a Predictor of Asymptomatic Hematogenous Metastasis in Gastric Cancer Patients
Source: PLoS One. 2014 Jul 1;9(7):e101125. doi: 10.1371/journal.pone.0101125 (PMC4077774; doi:10.1371/journal.pone.0101125)

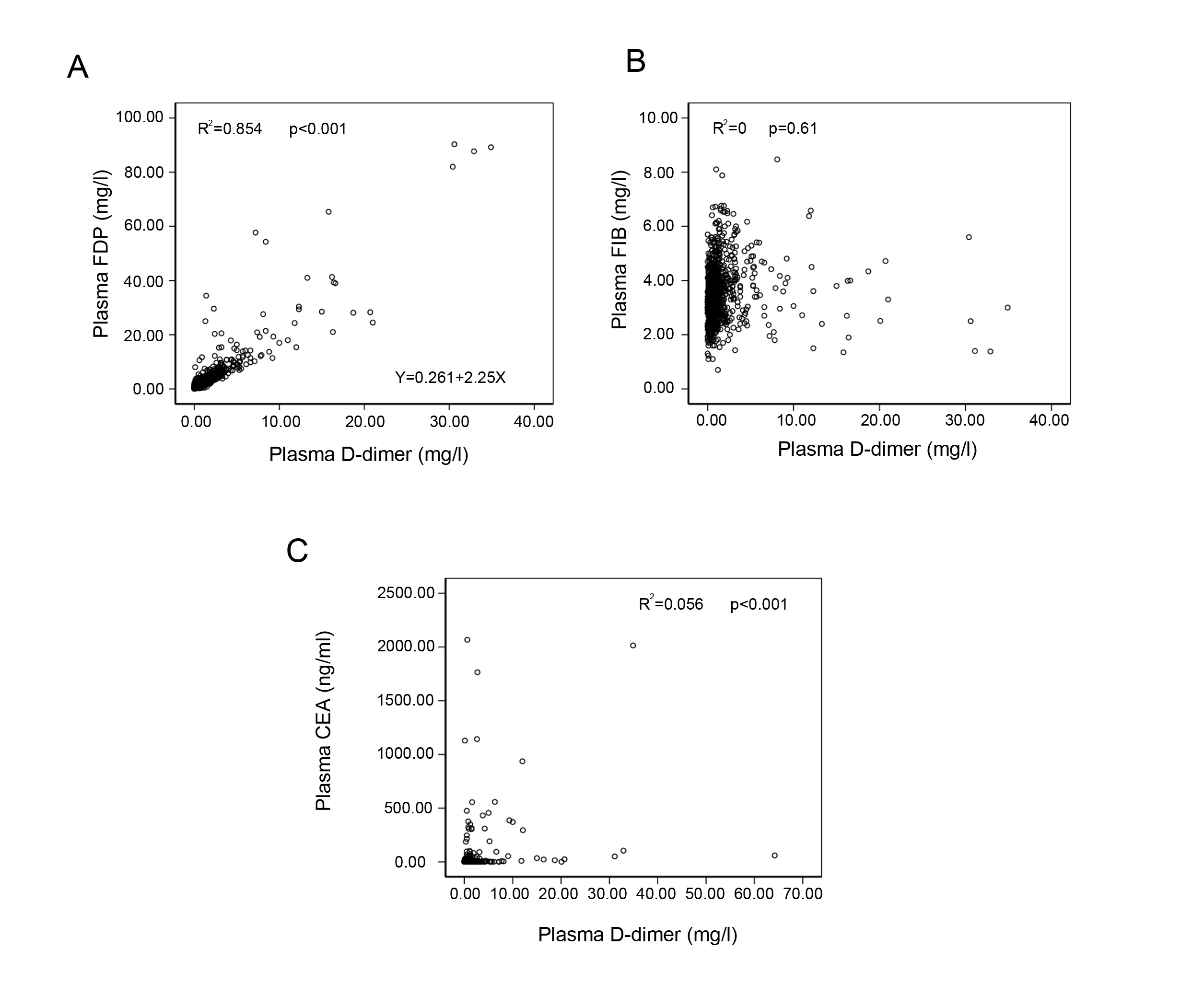

Supplement: Figure S1 — The relationship between plasma D-dimer levels and FDP, FIB and CEA. A, B, Plasma D-dimer levels showed a linear relation with plasma FDP levels but not with plasma FIB levels. C, Plasma D-dimer levels correlated with plasma CEA levels, although they did not display a linear relation. (TIF) [file pone.0101125.s001.tif]
