# Supplementary material for: D-Dimer: Not Just an Indicator of Venous Thrombosis but a Predictor of Asymptomatic Hematogenous Metastasis in Gastric Cancer Patients
Source: PLoS One. 2014 Jul 1;9(7):e101125. doi: 10.1371/journal.pone.0101125 (PMC4077774; doi:10.1371/journal.pone.0101125)
Supplement: Table S1 — Patient characteristics and plasma D-dimer (mg/l) levels in different groups. (DOC) [file pone.0101125.s002.doc]

Supplement Table 1 Patient characteristics and plasma D-dimer (mg/l) levels in different groups.

| Groups | No. case | Age,years, (median/(25^th^-75^th^ percentile)) | Gender (male/ female), n (%) | D-dimer ( Median /(25^th^-75^th^ percentile) ) |
| --- | --- | --- | --- | --- |
| Health | **50** | **59(52-66)** | **25 (50%) / 25( 50%)** | **0.80 (0.60- 1.10)** |
| GPs | **31** | **59(54-65)** | **10(32.3%)/ 21( 67.7%)** | **0.70 (0.20- 1.00)** |
| GSTs | **35** | **58(48-66)** | **23(65.7%)/ 12( 34.3%)** | **1.0 (0.3- 2.00)** |
| GC TNM stages | **1042** | **61(54-68)** | **837(80.3%)/ 205(19.7%)** | **0.70 (0.30-1.39)** |
| ⅠA | **74** | **60.5（54-68）** | **62( 83.8%)/ 12( 16.2%)** | **0.40（0.20-0.90）** |
| ⅠB | **61** | **58（53-64.5）** | **55( 90.2%)/ 6( 9.8%)** | **0.50（0.20- 1.19）** |
| Ⅱ | **178** | **61（54-68）** | **146(82.0%)/ 32( 18.0%)** | **0.50（0.24- 1.00）** |
| ⅢA | **259** | **61（55- 68）** | **219(84.6%)/ 40( 15.4%)** | **0.50（0.20- 1.00）** |
| ⅢB | **152** | **59.5（53-67）** | **121(79.6%)/ 31( 20.4%)** | **0.70（0.30-1.10）** |
| Ⅳ | **318** | **61（54-70）** | **234(73.6%)/ 84( 26.4%)** | **1.40（0.60-3.00）** |

Footnote: GP means gastric polyps; GC means gastric cancer; GSTs means gastric stromal tumors.

Supplement Table 2. Association between CEA levels (ng/ ml) and Clinicopathological Features in Patients with Gastric Cancer (n=364).

| Variables | *B* | *P* |
| --- | --- | --- |
| Gender | 0.025 | 0.699 |
| Age | 0.023 | 0. 711 |
| Histological grade | 0.049 | 0.453 |
| Tumor invasion | -0.015 | 0.831 |
| Lymph node invasion | 0.231 | 0.002** |
| Distant metastasis | 0.036 | 0.575 |
| Cancer embolus | -0.021 | 0.755 |

Footnote: B means partial regression confidence. P indicate that the p-value was analyzed via multivariate linear regression models ** indicates p-value ＜0.01 (2-tailed).
